# Establishment and validation of a nomogram for predicting new fractures after PKP treatment of for osteoporotic vertebral compression fractures in the elderly individuals

**DOI:** 10.1186/s12891-023-06801-3

**Published:** 2023-09-12

**Authors:** Yiming Ma, Qi Lu, Xuezhi Wang, Yalei Wang, Feng Yuan, Hongliang Chen

**Affiliations:** 1 Department of Orthopaedic Surgery, The Affiliated Hospital of Xuzhou Medical University, 99 Huaihai Road, Xuzhou, 221006 Jiangsu China; 2 Xuzhou Medical University, Xuzhou, 221004 Jiangsu China

**Keywords:** Osteoporosis, Vertebral kyphoplasty, Vertebral compression fracture, Nomogram, Prediction models

## Abstract

**Background:**

To investigate the risk factors for new vertebral compression fractures (NVCFs) after percutaneous kyphoplasty (PKP) for osteoporotic vertebral compression fractures (OVCFs) and to create a nomogram to predict the occurrence of new postoperative fractures.

**Methods:**

This was a retrospective analysis of the clinical data of 529 OVCF patients who received PKP treatment in our hospital from June 2017 to June 2020. Based on whether there were new fractures within 2 years after surgery, the patients were divided into a new fracture group and a nonnew fracture group. Univariate and multivariate analyses were used to determine the risk factors for the occurrence of NVCFs after surgery. The data were randomly divided into a training set (75%) and a testing set (25%). Nomograms predicting the risk of NVCF occurrence were created based on the results of the multivariate analysis, and performance was evaluated using receiver operating characteristic curves (ROCs), calibration curves, and decision curve analyses (DCAs). A web calculator was created to give clinicians a more convenient interactive experience.

**Results:**

A total of 56 patients (10.6%) had NVCFs after surgery. The univariate analysis showed significant differences in sex and the incidences of cerebrovascular disease, a positive fracture history, and bone cement intervertebral leakage between the two groups (*P* < 0.05). The multivariate analysis showed that sex [*OR* = 2.621, *95% CI* (1.030–6.673), *P* = 0.043], cerebrovascular disease [*OR* = 28.522, *95% CI* (8.749–92.989), *P* = 0.000], fracture history [*OR* = 12.298, *95% CI* (6.250–24.199), *P* = 0.000], and bone cement intervertebral leakage [*OR* = 2.501, *95% CI* (1.029–6.082),* P* = 0.043] were independent risk factors that were positively associated with the occurrence of NVCFs. The AUCs of the model were 0.795 (*95% CI*: 0.716–0.874) and 0.861 (*95% CI*: 0.749–0.974) in the training and testing sets, respectively, and the calibration curves showed high agreement between the predicted and actual states. The areas under the decision curve were 0.021 and 0.036, respectively.

**Conclusion:**

Female sex, cerebrovascular disease, fracture history and bone cement intervertebral leakage are risk factors for NVCF after PKP. Based on this, a highly accurate nomogram was developed, and a webpage calculator (https://new-fracture.shinyapps.io/DynNomapp/) was created.

## Introduction

The incidence of osteoporosis increases as the proportion of aging adults increases. Osteoporotic vertebral compression fractures (OVCFs) present with persistent low back pain, vertebral kyphosis, and decreased quality of life [1]. For patients with mild symptoms, pain can be relieved by conservative or pharmacological treatment, but many patients are unable to undergo nonsurgical treatment due to complications caused by braking [2]. In 1984, Galibert et al. first performed percutaneous vertebroplasty (PVP) for the treatment of haemangioma, and then in 1999, Mark Reiley, an American physician, improved PVP, thereby creating percutaneous kyphoplasty (PKP) [3]. PVP and PKP are minimally invasive procedures that rapidly relieve pain, immediately correct kyphotic deformities, and promote rapid postoperative recovery [4]. However, as the use of this technique becomes more widespread, its disadvantages are being gradually revealed. For example, bone cement leakage and new vertebral compression fractures (NVCFs) [5, 6]. Several studies have reported that the risk of NVCFs after PKP is between 5 and 50% [7–9]. Previous literature has reported that the risk factors for such fractures after PKP include age, sex, low BMI, low bone mineral density, positive fracture history, large Cobb angle, bone cement intervertebral leakage, presence of thoracolumbar fractures and poor vertebral height recovery [7–11]. NVCFs may require reoperation or conservative treatment, both of which can seriously affect the quality of life of the patient. Therefore, it is necessary to identify patients with a high risk of new postoperative fractures as early as possible to reduce the risk of their occurrence. Nomograms, which calculate the likelihood of clinical events by means of complex formulas, are increasingly used in various fields [12, 13]. With the help of nomograms, clinicians can assess the risk of clinical events, develop individualized treatment plans, and follow up more actively. To this end, we developed and validated a nomogram for predicting the occurrence of NVCFs by determining the pathogenic factors of NVCFs through a multivariate analysis in anticipation of better clinical feedback.

## Materials and methods

A total of 529 patients who underwent PKP to treat OVCFs at our institution from June 2017 to June 2020 were entered into the study cohort, and these patients were followed up for a mean duration of 28.92 ± 4.21 months.

### Inclusion and exclusion criteria

Inclusion criteria: (1) preoperative T2 image high signal of the fractured vertebrae confirmed by magnetic resonance imaging; (2) treated with PKP; (3) Bone mineral density T value  ≤ -2.5 on dual-energy X-ray; (4) follow-up time  > 2 years; (5) complete clinical and imaging data; and (7) complete follow-up data.

Exclusion criteria: (1) other causes of symptomatic low back pain (disc herniation, slipped vertebrae, lumbar isthmus fracture, etc.); (2) infectious disease; (3) benign or malignant tumours of the spine; (4) unstable fractures involving the posterior column; and (5) unwillingness to complete the follow-up.

### Surgical technique

All operations were performed by the same medical team. The patient was placed in a prone position, and the injured vertebra was located and marked using C-arm fluoroscopy before surgery. A 4-mm incision was made at the localization point under local anaesthesia, and a puncture needle (Suzhou Aide Technology Development Co., Ltd.) was placed along the vertebral arch under C-arm guidance, with an angle of approximately 15–20° between the needle and the sagittal plane of the body. The puncture needle was stopped when it reached the posterior anterior 1/3 of the vertebral body. A balloon was placed inside the vertebral body via the puncture needle core, and a contrast agent was injected to slowly expand the balloon. The extent of balloon expansion and the height of the vertebral body were observed under fluoroscopy, and when the balloon position was satisfactory, the balloon and contrast agent were removed. Polymethylmethacrylate (PMMA) bone cement (Tecres S.P.A.) was prepared in the form of toothpaste, 3–5 ml was injected into each vertebral body, and the distribution of the bone cement in the vertebral body was closely observed. The C-arm confirmed that the bone cement was well distributed, and the procedure was completed. PKP was performed on the opposite side using the same method as in the bilateral arch approach.

### Clinical follow-up

After surgery, the patients wore a support device around the waist to get out of bed and were advised to do muscle exercises while lying in bed to prevent deep vein thrombosis in the lower limbs. The frontal and lateral radiographs of the spine were reviewed 24 h after surgery. The patients attended follow-up visits at 1 month, 6 months, 1 year and 2 years postoperatively at the outpatient clinic. If the patient did not return for the follow-up visit, they were called and asked to disclose the reason for missing the follow-up visit. If the patient suddenly developed back pain during the follow-up period, MRI was performed to determine if a new vertebral fracture had occurred. Diagnostic criteria for the development of NVCFs after PKP were as follows: 1) reappearance of low back pain after postoperative pain relief and limited movement, especially when turning or getting up. 2) MRI showing a high T2 signal and a low T1 signal. Postoperatively, if there was no contraindication, calcium carbonate D3 (Jiangsu Suzhou Wyeth Pharmaceutical Co., Ltd., orally, 1 time/day, 1 tablet/time, for 2–3 months), osteoporotic triol (Shandong Qingdao Zhengda Pharmaceutical Co., Ltd., orally, 1 time/day, 0.25 µg/day, for 2–3 months), and zoledronic acid injection (Novartis, Switzerland, intravenous infusion, 5 mg/dose once/year for 2–3 years) were used to treat osteoporosis. Patients were asked at each review whether they complied with the doctor's orders for anti-osteoporosis treatment.

### Observation indicators

The following information was recorded preoperatively.

(1) General information: sex, age, body mass index (BMI), chronic diseases (hypertension, diabetes, respiratory diseases, heart diseases and cerebrovascular diseases), fracture history (previous fracture of any part of the body), time of injury, time from admission to surgery, location of fractured vertebrae, number of fractured vertebrae, and type of fracture. (2) Surgical factors: surgical approach (unilateral or bilateral), new vertebral fracture, type of cement leakage (paravertebral leakage, intervertebral leakage, spinal leakage), cement distribution, cement-to-endplate contact, vertebral height recovery rate, and postoperative Cobb angle.

Fracture history: history of other vertebral fractures (with or without symptoms) that occurred prior to the OVCF or a history of fractures elsewhere in the body. Old vertebral fractures on magnetic resonance images were also indicative of a positive history of prior fracture.

Bone cement distribution: bone cement not crossing the midline of the vertebral body on the frontal X-ray was defined as unilateral distribution; otherwise, it was bilateral distribution; if the bone cement was discontinuous bilaterally, it was bilaterally separated distribution; otherwise, it was bilaterally fused distribution.

Vertebral height recovery rate: the anterior edge (biconcave fracture) or midline (wedge fracture) height of the fractured vertebrae was recorded preoperatively and 24 h postoperatively (the height of the anterior edge of each fractured vertebra was taken, and the average value was calculated). In this study, the average value of the heights of the two adjacent normal, same-segment vertebrae of the fractured vertebrae was taken as the normal vertebral height, and then the vertebral height recovery rate was calculated with the following formula: normal vertebral height H0 = (previous normal vertebral height H1 + next normal vertebral height H2)/2. Vertebral body height recovery rate = (postoperative height of injured vertebra—preoperative height of injured vertebra)/H0 * 100%.

Cobb angle: the angle between the upper edge of the head end of the fractured vertebral body and the lower edge of the tail end.

### Statistical analyses

Continuous variables were expressed as the mean ± standard deviation, and categorical variables were expressed as ratios. The data were randomly divided into a training set (75%) and a validation set (25%). In this study, the training set was used to construct a nomogram, and the testing set was used to validate the efficacy of the nomogram.

Univariate and multivariate logistic regression analyses were used to filter the variables in the dataset, and variables with *P* < 0.05 were included in the model. The "rms" package in R-Studio was used to build the nomogram. Calibration curves of the model were drawn using the 1000-sample validation method to determine the consistency of the model. The predictive power of the nomogram was tested using tenfold cross-validation. The sensitivity and specificity of the model were evaluated using the area under the curve (AUC), and the larger the AUC value, the better the predictive power of the model. Decision curve analysis (DCA) was performed to assess the clinical utility of the model. The model capability was further validated in the testing set following the same method as above.

Data analysis was performed using SPSS (Version 26.0, IBM Corporation, Chicago, USA) and R-Studio (Version 3.6.2, R Foundation for Statistics Computing, Vienna, Austria), and several R packages were applied, including regplot, rms, ggDCA, ggplot, pROC, etc., to plot nomograms, calibration plots, decision curves, and ROC curves. *P* < 0.05 was statistically significant.

## Results

### Basic information

**Fig. 1:**
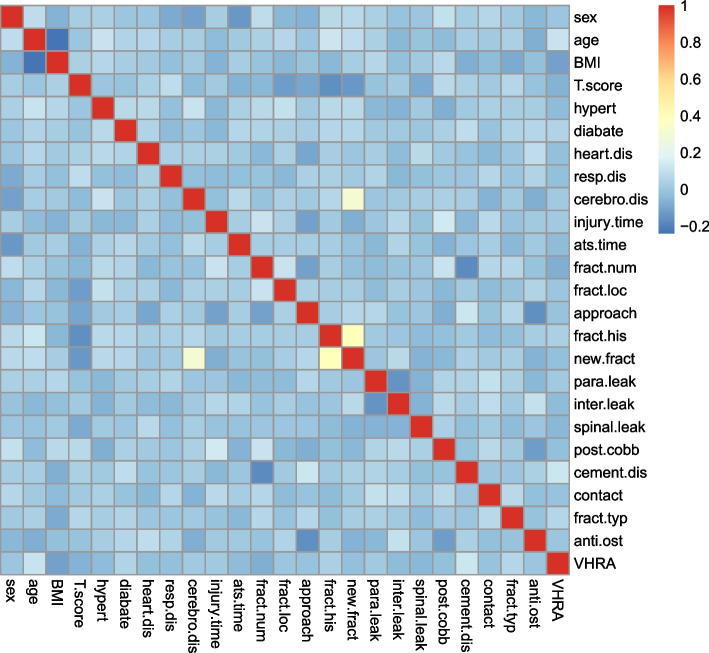
Heatmap of Data Correlation. Factors near positive colors are highly expressed and positively correlated, while factors near negative colors are lowly expressed and negatively correlated. Each square indicates the correlation between the factors in that row and column, and the color is used to indicate the amount of correlation. Abbreviation: hypert (hypertension); heart.dis (heart disease); resp.dis (respiratory diseases); cerebro.dis(cerebrovascular disease); ats.time(time from hospital admission to surgery); fract.num (fracture number); fract.loc (fracture location); fract.his (fracture history); new.fract (new fracture); para.leak (paravertebral leakage); inter.leak (intervertebral leakage); spinal.leak (spinal leakage); post.cobb (post-operation cobb angle); cement.dis (cement distribution); fract.typ (fracture type); anti.ost (anti-osteoporosis); VHRA (vertebral height recovery rate)

The clinical data of 529 patients, including 135 males and 394 females, with a mean age of 71.185 ± 10.012 years, were included in this study. Among them, 56 patients had new fractures after surgery, and 473 patients did not have any new fractures. The patients’ baseline data are presented in Table 1. Figure 1 shows the heatmap of the correlation of the dataset.

**Table 1:** Baseline information [n (%), x ® ± s]

Baseline information	
Patients (n)	529
Age (years)	71.185 ± 10.012
Sex (M/F)	135 (25.5)/394 (74.5)
BMI (kg/m^2^)	23.394 ± 3.299
BMD	-2.811 ± 0.270
Hypertension	200 (37.8)/329 (62.2)
Diabetes	45 (8.5)/484 (91.5)
Heart disease	28 (5.3)/501 (94.7)
Respiratory diseases	7 (1.3)/522 (98.7)
Cerebrovascular disease	21 (4.0)/508 (96.0)
Time of injury	26.718 ± 67.426
Time from admission to surgery	3.010 ± 2.552
Number of fractured vertebral bodies	1.327 ± 0.693
Location of the fractured vertebral thoracic/thoracolumbar spine/lumbar	72 (13.6)/347 (65.6)/110 (20.8)
Operation approach unilateral/bilateral	147 (27.8)/382 (72.2)
Fracture history (Y/N)	90 (17.0)/439 (83.0)
New fracture (Y/N)	56 (10.6)/473 (89.4)
Paravertebral leakage	74 (14)/455 (86)
Intervertebral leakage	63 (11.9)/466 (88.1)
Spinal leakage	12 (2.3)/517 (97.7)
Cobb of post operation	12.508 ± 8.293
Bone cement distribution unilateral/bilateral fusion/bilateral separated	119 (22.5)/285 (53.9)/125 (23.6)
Bone cement contact with the endplate	417 (78.8)/417 (21.2)
Fracture type biconcave/wedge/compression	208 (39.3)/264 (49.9)/57 (10.8)
Anti-osteoporosis (Y/N)	429 (81.1)/429 (18.9)
Vertebral height recovery height	14.393 ± 13.441

### Univariate and multivariate analyses

The univariate analysis (Table 2) showed statistically significant differences (*P* < 0.05) in age, BMD, cerebrovascular disease status, and fracture history between the two groups. The multivariate analysis showed (Table 2) that sex [*OR* = 2.621, *95% CI* (1.030–6.673), *P* = 0.043], cerebrovascular disease [*OR* = 28.522, *95% CI* (8.749–92.989),* P* = 0.000], fracture history [*OR* = 12.298, *95% CI* (6.250–24.199), *P* = 0.000], and cemented intervertebral leakage [*OR* = 2.501, *95% CI* (1.029–6.082), *P* = 0.043] were independent risk factors positively associated with new fractures.

**Table 2:** Univariate and multivariate analysis

Variables	Univariate analysis	*P*	Multivariate analysis	*P*
Age	1.896 (0.903–3.981)	0.091	/	/
Sex	1.032 (1.001–1.064)	0.041	2.621(1.030–6.673)	0.043
BMI	1.029 (0.947–1.119)	0.498	/	/
BMD	0.235 (0.091–0.609)	0.003	/	/
Hypertension	1.615 (0.925–2.817)	0.092	/	/
Diabetes	1.964 (0.865–4.460)	0.107	/	/
Heart disease	1.415 (0.167–11.972)	0.750	/	/
Respiratory diseases	1.014 (0.296–3.473)	0.982	/	/
Cerebrovascular disease	14.061 (5.615–35.208)	0.000	28.522(8.749–92.989)	0.000
Time of injury	0.985 (0.970–1.001)	0.070	0.985(0.969–1.001)	0.065
Time from admission to surgery	0.995 (0.892–1.111)	0.931	/	/
Number of fractured vertebral	0.926 (0.607–1.414)	0.722	/	/
Location of the fractured vertebral	1.192 (0.740–1.920)	0.470	/	/
Operation approach	1.651 (0.829–3.287)	0.154	/	/
Fracture history	10.471 (5.747–19.081)	0.000	12.298(6.250–24.199)	0.000
New fracture	1.028 (0.465–2.270)	0.946	/	/
Paravertebral leakage	1.979 (0.964–4.063)	0.063	/	/
Intervertebral leakage	0.000 (0.000-inf)	0.983	2.501(1.029–6.082)	0.043
Spinal leakage	0.982 (0.947–1.018)	0.311	/	/
Bone cement distribution	1.209 (0.802–1.821)	0.364	/	/
Bone cement in contact with the endplate	1.111 (0.554–2.225)	0.767	/	/
Fracture type	1.151 (0.754–1.757)	0.515	/	/
Anti-osteoporosis(Y/N)	0.667 (0.349–1.275)	0.220	/	/
Vertebral height recovery height	0.995 (0.974–1.017)	0.660	/	/

**Fig. 2:**
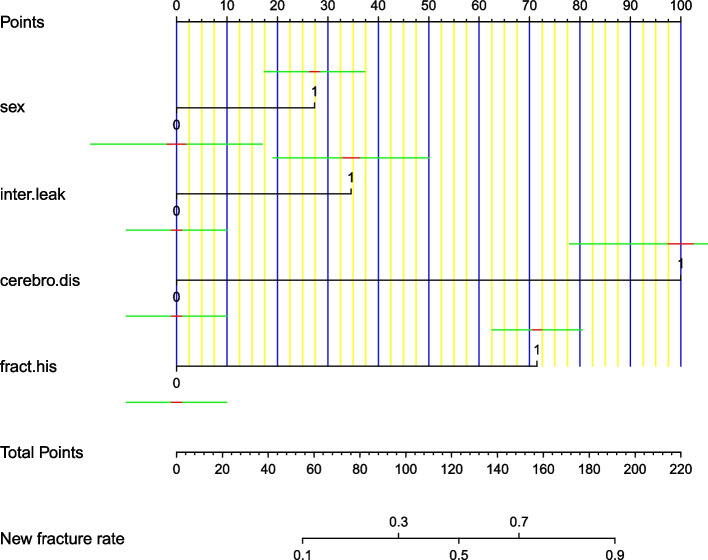
Nomogram. Abbreviations: inter.leak: intervertebral leakage; cerebro.dis: cerebrovascular disease; frac.his: fracture history

**Fig. 3:**
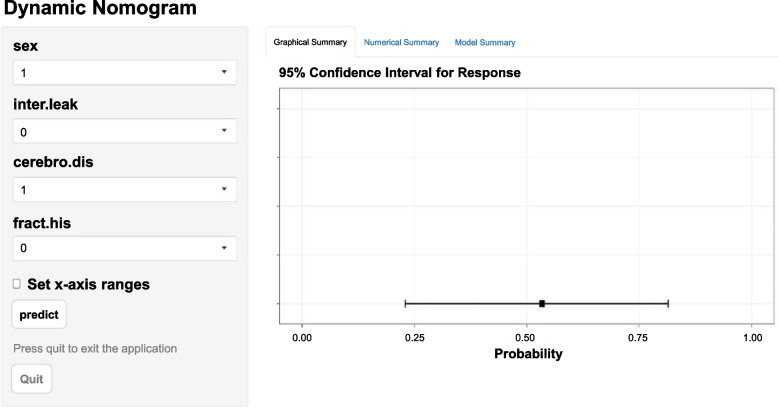
web calculator. A web-based interactive calculator interface based on a nomogram for predicting the risk of NVCF after PKP. Assignment method: sex: 0, male; 1, female; inter.leak (intervertebral space leakage): 0 (no), 1 (yes); cerebro.dis (cerebrovascular disease): 0 (no), 1 (yes); fract.his (fracture history): 0 (no), 1 (yes)

Independent predictors were derived by multivariate analysis, and four predictors were finally included in the model: female sex, positive fracture history, cerebrovascular disease diagnosis, and cemented intervertebral leakage (Fig. 2). A web calculation (Fig. 3) was created based on the results of the study (https://new-fracture.shinyapps.io/DynNomapp/). Each factor in the nomogram corresponds to the score of the vertex axis, and finally, the scores of each factor were summed to calculate the total score. A straight line was drawn from the corresponding total score point to obtain the outcome probability.

**Fig. 4:**
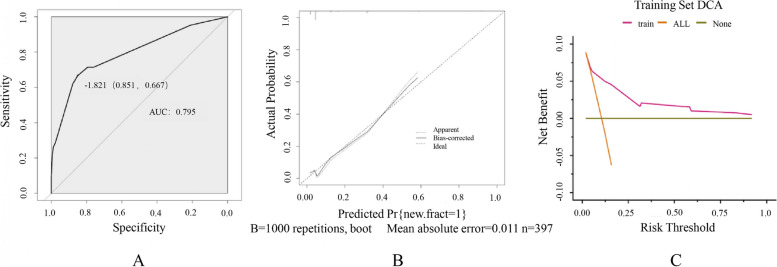
ROC curve, calibration curve and decision curve of training set. **A** ROC curve; **B** calibration curve; **C** DCA curve

**Fig. 5:**
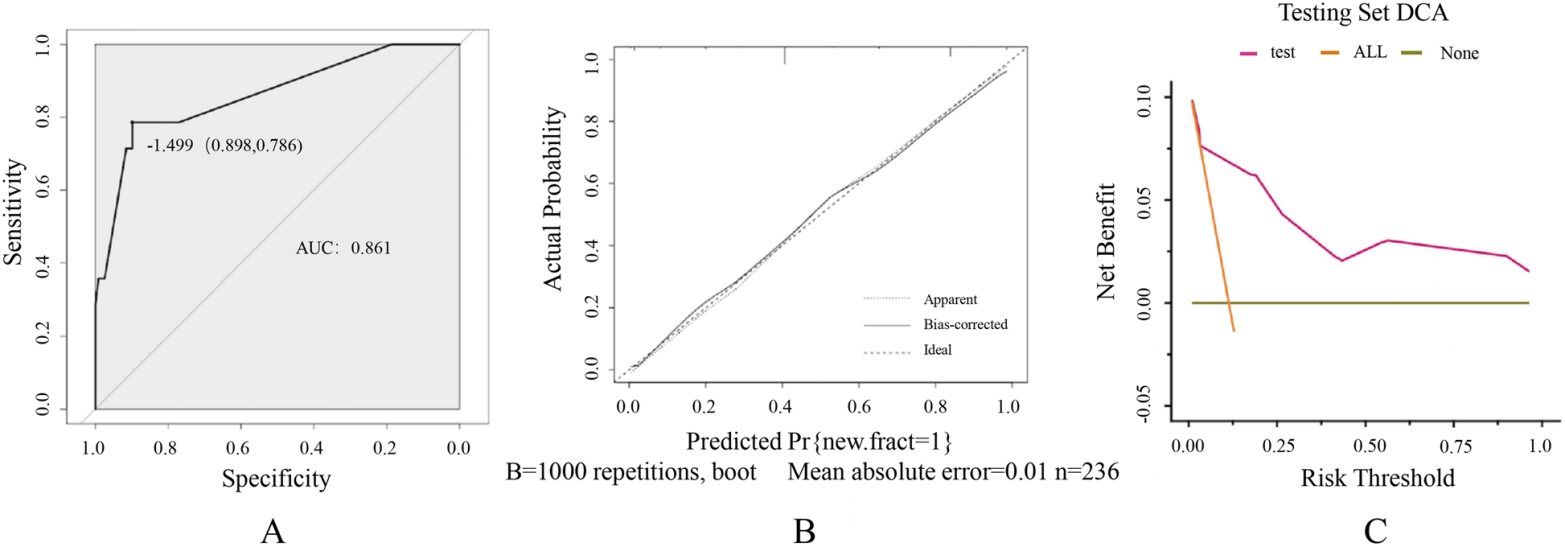
ROC curve, calibration curve and decision curve of testing set. **A** ROC curve; **B** calibration curve; **C** DCA curve

The model was validated by AUC, calibration curves, and decision curves. In the training set, the ROC curve showed that the obtained model had a good discriminatory ability with an AUC of 0.795 (*95% CI*: 0.716–0.874), indicating that it could predict the risk of NVCF development after PKP more accurately. The calibration curve showed high agreement between the prediction of the nomogram and the actual observation (Fig. 4), and the area under the decision curve (AUDC) was 0.021. In the testing set, the AUC of the model was 0.861 (*95% CI*: 0.749–0.974), the calibration curve assessed good agreement between the predicted and observed actual results, and the AUDC was 0.036 (Fig. 5).

## Discussion

As a minimally invasive technique, PKP has been widely used in clinical practice in recent years. NVCFs are one of the most common complications after surgery for OVCFs. Nomograms are a visual prediction tool based on a statistical regression model that can measure the influence of various factors on the possibility of event occurrence and have been widely used in various medical fields [12–14].

There are many factors affecting the development of NVCFs. The multivariate analysis revealed that female sex, cerebrovascular disease diagnosis, positive fracture history, and intervertebral leakage of bone cement were independent risk factors for NVCFs after surgery for OVCFs. Based on this, we developed a nomogram based on four of the more influential and readily available independent predictors to provide an accurate tool to predict new postoperative fracture risk. At the same time, the results of the internal validation also show good discriminative and calibration abilities, and the higher AUC value indicates that the nomogram can be widely and accurately applied.

Oestrogen can directly affect bone metabolism by regulating cellular physiological functions. The decrease in oestrogen levels in postmenopausal women inevitably leads to the weakening of its inhibitory effect on osteoclasts, an increase in the number of osteoclasts, a decrease in apoptosis, and the prolongation of the lifespan, which enhances bone resorption and promotes the progression of osteoporosis. Although osteoblast-mediated bone formation was also increased, it was not sufficient to compensate for excessive bone resorption. Active and unbalanced bone remodelling leads to thinning or fracture of trabecular bone, increased cortical bone porosity leads to decreased bone strength, and decreased oestrogen reduces bone sensitivity to mechanical stimulation, resulting in bone exhibiting pathological changes such as disuse bone loss [15]. A multicentre large-sample cohort study on the prevalence of osteoporosis in Chinese individuals by Zeng et al. [16] found that the number of women suffering from osteoporosis is much greater than that of men. In the United States, approximately 1 in 2 white women or 1 in 5 men will experience an osteoporosis-related fracture in their lifetime [17]. However, a large cross-sectional study by Wang et al. [18] found that in China, 5.0% of men and 20.6% of women aged 40 or older had osteoporosis, and 10.5% of men and 9.7% of men aged 40 or older had vertebral fractures. The similar prevalence of vertebral fractures in men and women suggests that we should also pay attention to the prevention and treatment of osteoporosis in men.

A multivariate analysis showed that the presence of cerebrovascular disease [*OR* = 28.522, *95% CI* (8.749–92.989), *P* = 0.000] was associated with a higher risk of postoperative NVCFs. A study by Tanislav et al. [19] showed that the occurrence of stroke as well as transient cerebral ischaemia was positively associated with fracture. Various adverse outcomes, such as depression, pain and reduced quality of life following stroke occurrence, lead to a higher risk of falls and fractures [20]. A large cohort study by Wang et al. [21] found that patients had a more than 8% risk of fracture 5 years after stroke occurrence and that stroke was significantly associated with fracture risk. Stroke in certain vascular regions of the brainstem can lead to impaired body balance and an increased risk of falls [22]. In addition, impairment of visual, motor, sensory or cognitive function after the onset of cerebrovascular disease may also lead to fall-related injuries [23]. Within 2 years after stroke, 60.7% of individuals who fell once experienced a second or subsequent fall, and 23.4% of patients had a fracture [24]. In addition to falls, the accelerated decrease in bone mineral density after stroke may lead to fractures in stroke patients [25]. Poststroke muscle weakness leads to limited weight bearing and reduced activity of the limb, which results in reduced bone mass. In addition, malnutrition, reduced sun exposure, and vitamin D deficiency can exacerbate bone loss in stroke survivors. Common stroke treatments, such as oral anticoagulants, can also increase the risk of osteoporosis and fracture [26]. Therefore, effective measures should be taken for skeletal health screening and fracture prevention in patients with cerebrovascular disease.

Osteoporosis progresses slowly, is not easily detected and may remain unnoticed for years until patients develop painful symptoms. In this study, most patients were first diagnosed with osteoporosis because of symptomatic vertebral fractures. Patients with previous fractures will therefore be at significantly increased risk of refracture in the future [27]. In the present study, 90 (17.0%) patients had a history of previous fracture. Regression analysis showed that the presence of a history of previous fracture was a high-risk factor for new fractures after PKP surgery. A history of previous fracture increased the risk of new fractures after surgery 12.298-fold [*OR* = 12.298, *95% CI* (6.250–24.199), *P* = 0.000]. This result suggests that a history of previous fractures is an important factor in the occurrence of new fractures after PKP.

When vertebral compression severely involves the endplate or is due to improper puncture, it can lead to the leakage of bone cement through the ruptured endplate to the intervertebral disc, thus altering the surrounding stresses [28]. A study by Nieuwenhuijse et al. [29] found a significant association between the leakage of bone cement to the intervertebral disc and the occurrence of postoperative NVCFs. The multifactorial analysis in our study showed that the leakage of bone cement into the intervertebral disc [*OR* = 2.*501, 95% CI* (1.029–6.082), *P* = 0.043] was a risk factor that was positively associated with the occurrence of NVCFs. The altered stiffness of the vertebral body after consolidation of the injured vertebral body and the cushioning effect of an otherwise intact disc can reduce the impact on the adjacent vertebral body, but when the bone cement leaks into the disc, it can increase the stress on the endplate of the adjacent vertebral body, and this alteration may increase the risk of NVCFs [30]. In addition, the heat generated by the bone cement leaking into the disc may cause some damage to the disc, which may also be a major contributor to accelerated disc degeneration [31].

In this study, we established a nomogram model based on a larger cohort and successfully validated the model in a validation cohort. Each variable included in the nomogram is a relatively accessible factor. By calculating scores for each of the 4 factors, orthopaedic surgeons can easily estimate the risk of NVCFs after surgery. Based on the assessment results, patient management strategies can be improved to reduce the risk of NVCFs. Likewise, for low-risk patients, some preventive measures can be implemented to reduce the financial burden.

This study has some limitations. First, this was a retrospective study, so there may be selection bias. However, we included as many preoperative and surgical factors because of the large sample of patients to minimize bias. Second, this study is a single-centre study. Although this nomogram has been validated in a validation cohort, the incidence of postoperative refractures varies across hospitals, regions, and countries, which may limit the application of this model in some hospitals. Third, 1 of the 4 parameters included in the model was determined postoperatively, which may not better assess the likelihood of postoperative refracture in patients preoperatively. Through multicentre retrospective studies or prospective randomized clinical trials, the sensitivity and specificity of nomograms can be further improved, providing high-level evidence for future clinical applications.

## Conclusion

Female sex, cerebrovascular disease diagnosis, positive fracture history and bone cement intervertebral leakage are risk factors for NVCFs after PKP. Based on this, a highly accurate nomogram was developed, and a webpage calculator (https://new-fracture.shinyapps.io/DynNomapp/) was created.

## Data Availability

The original data in the study will be made available by the authors, further inquiries can be directed to the corresponding author.
